# Uterine Microbiota and Immune Parameters Associated with Fever in Dairy Cows with Metritis

**DOI:** 10.1371/journal.pone.0165740

**Published:** 2016-11-01

**Authors:** Soo Jin Jeon, Federico Cunha, Xiaojie Ma, Natalia Martinez, Achilles Vieira-Neto, Rodolfo Daetz, Rodrigo C. Bicalho, Svetlana Lima, Jose E. P. Santos, K. Casey Jeong, Klibs N. Galvão

**Affiliations:** 1 Department of Large Animal Clinical Sciences, College of Veterinary Medicine, University of Florida, Gainesville, Florida, United States of America; 2 Department of Animal Sciences, University of Florida, Gainesville, Florida, United States of America; 3 Department of Population Medicine and Diagnostic Sciences, Cornell University, Ithaca, New York, United States of America; 4 Emerging Pathogens Institute, University of Florida, Gainesville, Florida, United States of America; 5 D. H. Barron Reproductive and Perinatal Biology Research Program, University of Florida, Gainesville, Florida, United States of America; Massachusetts General Hospital, UNITED STATES

## Abstract

**Objective:**

This study aimed to evaluate bacterial and host factors causing a fever in cows with metritis. For that, we investigated uterine microbiota using a metagenomic sequencing of the 16S rRNA gene (Study 1), and immune response parameters (Study 2) in metritic cows with and without a fever.

**Principal Findings (Study1):**

Bacterial communities were similar between the MNoFever and MFever groups based on distance metrics of relative abundance of bacteria. Metritic cows showed a greater prevalence of Bacteroidetes, and *Bacteroides* and *Porphyromonas* were the largest contributors to that difference. A comparison of relative abundance at the species level pointed to *Bacteroides pyogenes* as a fever-related species which was significantly abundant in the MFever than the MNoFever and Healthy groups; however, absolute abundance of *Bacteroides pyogenes* determined by droplet digital PCR (ddPCR) was similar between MFever and MNoFever groups, but higher than the Healthy group. The same trend was observed in the total number of bacteria.

**Principal Findings (Study2):**

The activity of polymorphonuclear leukocyte (PMN) and the production of TNFα, PGE_2_ metabolite, and PGE_2_ were evaluated in serum, before disease onset, at 0 and 3 DPP. Cows in the MNoFever had decreased proportion of PMN undergoing phagocytosis and oxidative burst compared with the MFever. The low PMN activity in the MNoFever was coupled with the low production of TNFα, but similar PGE_2_ metabolite and circulating PGE_2_.

**Conclusion/Significance:**

Our study is the first to show a similar microbiome between metritic cows with and without a fever, which indicates that the host response may be more important for fever development than the microbiome. *Bacteroides pyogenes* was identified as an important pathogen for the development of metritis but not fever. The decreased inflammatory response may explain the lack of a febrile response in the MNoFever group.

## Introduction

Metritis is an inflammatory disease in the uterus within 21 days after parturition that affects the cow’s welfare and leads to decreased milk yield, decreased reproductive performance, and economic loss [1–3]. It has been proposed that cows with an abnormally enlarged uterus, a fetid watery red-brownish uterine discharge, and a fever be classified as puerperal metritis, whereas the term, metritis should be used for cows with delayed uterine involution and a fetid discharge in the absence of a fever [4]. In cows with fetid watery red-brownish uterine discharge, 40–60% do not have a fever [5,6]. Nonetheless, only cows with a fever have been evaluated for approval of new antibiotics for treatment of metritis [7,8]. Metritic cows without a fever has been reported to have the same decrease in milk yield [9] and increase in clinical endometritis [5] as metritic cows with a fever; however, a decrease in reproductive performance was only observed in metritic cows with a fever [9]. Furthermore, metritic cows without a fever presented a greater cure rate after treatment than did metritic cows with a fever [10]. The difference in cure rate could be due to differences in the microbiota associated with fever, which could affect the response to antibiotic treatment.

A key event in initiating an inflammatory response and a fever is the induction and release of endogenous cytokines (i.e., TNF*α*, IL-1*β*, IL-6) and chemokines (i.e., RANTES, MCP-1) by polymorphonuclear and mononuclear leukocytes, which ultimately leads to synthesis of prostaglandin E_2_ (PGE_2_) and triggers a fever in the central nervous system. [11–13]. Both lipopolysaccharide (LPS) from Gram-negative bacteria or peptidoglycans from Gram-positive bacteria can induce a fever, but LPS has been shown to be more potent in rodent models [13]. In cows with mastitis, infections with Gram-negative pathogens have also been shown to be more severe [14]. We recently observed that the uterine microbiota of metritic cows with a fever was dominated by Gram-negative bacteria such as Bacteroidetes and Fusobacteria [15]; therefore, the uterine microbiota could influence the development of fever.

Neutrophils are the main phagocyte involved in propagating the inflammatory response and in bacterial clearance during uterine infection [16,17]. Impairment of neutrophil function was observed in metritic cows starting 1 week before calving, and continuing until 3 weeks after calving [18]. Thus, the immunosuppression of pre- and post- partum dairy cows may be a determinant for the development of metritis given that all cows are naturally infected with bacteria during or shortly after parturition but not all cows develop disease [15]. Indeed, a previous study reported a decrease in the neutrophil function around the time of calving in cows that developed metritis [17–19]. Nonetheless, there has been no study differentiating immune response between metritic cows with and without a fever. Therefore, an alternate hypothesis to the bacterial influence on fever development is that the immune response to the bacterial challenge could be the main determinant of fever in metritic cows.

Because fever is usually interpreted as a sign of severity of the infection, and was an important determinant of cure rate and reproductive performance of dairy cows with metritis, we sought to identify uterine microbiota and immune parameters associated with fever in metritic cows. Our initial hypothesis was that metritic cows with a fever would have a higher abundance of gram-negative bacteria and/or a greater bacterial load than metritic cows without a fever. After observing that the microbial composition and bacterial load was very similar between metritic cows with and without a fever, we set out to explore immune parameters that could contribute to fever development or lack thereof in metritic cows. Therefore, first we compared bacterial communities from postpartum cows depending on health status using a high throughput sequencing of 16S rRNA gene on the Illumina MiSeq platform. To identify fever-related bacteria, we compared relative abundance of bacterial species among groups. For validation of metagenomic analysis, absolute quantification was also performed using droplet digital PCR (ddPCR). Then, to describe immune response of cows in terms of fever induction, we examined phagocytic activity and oxidative burst of polymorphonuclear leukocyte (PMN) as well as the production of TNFα and PGE_2_ metabolite in serum samples.

## Materials and Methods

### Animal management

All animal procedures were approved by the University of Florida Institutional Animal Care and Use Committee (no. 201207405). Two hundred and two Holstein cows from a commercial dairy in Central Florida milking 5,000 cows were used in this study. Animals enrolled in this study are presented in S1 Table. Metadata for study 1 and study 2 are described in S2 Table.

### Study 1—diagnosis of metritis, rectal temperature, and sampling for uterine microbiota analysis

Metritis was diagnosed by examining the uterine discharge from 92 cows at 4, 6, and 8 days postpartum (6 ± 2 DPP) using a 5-point scale as previously described [15]: 1 = not fetid normal lochia, viscous, clear, red, or brown; 2 = cloudy mucoid discharge with flecks of pus; 3 = not fetid mucopurulent discharge with < 50% pus; 4 = not fetid mucopurulent discharge with ≥ 50% pus; 5 = fetid red-brownish, watery discharge. Cows with a discharge score ≤ 4 were classified as healthy (n = 40) and cows with a score of 5 were classified as metritic cows. Rectal temperature (RT) was measured at calving and at 2, 4, 6, and 8 DPP. Cows with RT ≥ 39.5°C were considered to have a fever. Metritic cows were grouped according to presence of fever at the time of metritis diagnosis; cows were classified as with (n = 23) or without a fever (n = 29).

Uterine swab samples were collected at the time of uterine discharge evaluation at 4, 6 and 8 DPP, and swab samples collected at the day of metritis diagnosis were used for metagenomic sequencing. All samples were collected prior to antibiotic treatment. The farm personnel checked all cows daily from 0 to 3 DPP; therefore, any cows diagnosed with metritis and treated with antibiotics before 4 DPP were not used for this study. Uterine swabs were collected using a 30” double-guarded sterile culture swab (Continental Plastics Corporation, Delavan, WI). The instrument was gently passed through the cervix and positioned in the uterine body where the internal sheath and the swab were exposed, and the swab was gently rolled against the uterine wall. The swab was retracted within the double sheath before removal from the cow. Uterine swab samples from 12 cows that developed metritis but did not have a fever at the time of metritis diagnosis (MNoFever) were randomly selected for metagenomic sequencing. The 16S data for metritic cows with a fever (MFever; n = 11) and healthy cows (Healthy; n = 11) were obtained from a previous study [15], and were used as reference groups for comparison with the MNoFever group. In the previous study, 12 samples from MFever and 12 from Healthy cows had been randomly selected; however, one sample from each group failed to amplify. Therefore, herein, 12 samples from the MNoFever were also selected to allow for the loss of at least one sample and still maintain similar group sizes. S1 Fig shows RT in postpartum cows from -6 to 4 days relative to diagnosis for cows used in the 16S metagenomic analysis. Cows from the MFever group had a fever from -2 to 4 days relative to metritis diagnosis, while cows from the Healthy and MNoFever groups did not develop a fever during the experiment.

#### Genomic DNA extraction

Frozen swabs were soaked and incubated in 1 ml of phosphate-buffered saline (PBS) on ice for 2 h. During incubation the swabs in PBS were repeatedly vortexed to maximize the release of uterine bacteria from the swabs. After incubation the swabs were discarded and PBS suspension containing bacteria was used to isolate the genomic DNA (gDNA) of uterine bacteria from dairy cows. All samples were pre-incubated with 500 μg of lysozyme at 37°C for 1 h to degrade the peptidoglycan layer of the cell wall of Gram-positive bacteria [20], and then gDNA of all bacteria was extracted using the QIAamp DNA Mini kit (Qiagen, Valencia, CA, USA) according to the manufacturer’s instruction. Although lysozyme treatment is a common step before gDNA extraction [21,22], bead beating and/or mutanolysin have been shown to produce significantly better bacterial community structure representation than lysozyme methods [23]; therefore, should be used in the future. The purity and concentration of gDNA were measured with a spectrophotometer (Nanodrop 2000, Thermo Scientific, Waltham, MA, USA).

#### PCR amplification and sequencing

We performed 16S amplicon sequencing according to a previously described method [24]. DNA template was tagged with 12-bp error-correcting Golay barcodes and the V4 hypervariable region of the bacterial 16S rRNA gene was amplified using 10 μM of primer 515F and 806R, 1× GoTaq Green Master Mix (Promega, Madison, WI, USA), 1 mM MgCl_2_, and the 5'-barcoded amplicons in triplicate. PCR was carried out as follows: an initial denaturing step at 94°C for 3 min, followed by 35 cycles of 94°C for 45 s, 50°C for 1 min, and 72°C for 90 s, and a final elongation step at 72°C for 10 min. Replicate amplicons were pooled and purified with a QIAquick PCR Purification Kit (Qiagen). The purified amplicons were standardized to the same concentration using Qubit^®^ 3.0 Fluorometer (Thermo Fisher Scientific), and sequencing was conducted on the Illumina MiSeq platform (Illumina, Inc., San Diego, CA, USA) using the MiSeq reagent kit v2-300 cycles. None of the samples from the MNoFever group failed to amplify; therefore 34 samples were used for data analysis. Taxonomy (phylum, order, class, family, genus, and species) and relative abundance were identified using the MiSeq Reporter Proprietary Metagenomics Workflow based on the Greengenes database (http://greengenes.lbl.gov). Metagenome sequences were deposited in the Metagenomics RAST (MG-RAST, http://metagenomics.anl.gov) under the ID numbers listed in S3 Table.

#### Droplet digital PCR

Droplet digital PCR (ddPCR) was performed on the QX200 system (Bio-Rad, Hercules, CA, USA) using DNA binding dye (EvaGreen) according to the manufacturer’s instruction. Primer set targeting *Bacteroides pyogenes* (F, 5'-GAAACGACAACCGGAGGTAA-3'; R, 5'-GTCAGCTTTCCAAGCACCTC-3') was designed in species-specific region in *recA* gene using Primer3 version 4.0.0 [25]. Total bacterial load was also assessed using the 16S universal primers [26]. To ensure primer specificity, primer pairs were tested in Primer-BLAST (Basic Local Alignment Search Tool) and melting temperatures were evaluated using qPCR (Applied Biosystems^®^ 7500 Fast). The ddPCR reaction mixture consisting of 10 μl Supermix (Bio-Rad), 250 nM primers, and gDNA isolated from uterine swab samples (1 ng) in a final volume of 20 μl was mixed with 20 μl of droplet generation oil (Bio-Rad) and partitioned into approximately 20,000 droplets in the QX200 droplet generator (Bio-Rad). The droplets generated from each sample were transferred to a 96-well plate and PCR amplification was conducted in the PTC-100 (Bio-Rad) with the following condition: 95°C for 10 min, 40-cycles of 94°C for 30 sec, 55°C for 45 sec, and 72°C for 50 sec, followed by 72° for 5 min and a hold at 4°. After amplification, the 96-well plate was loaded onto the QX200 droplet reader (Bio-Rad) which automatically measures the fluorescence intensity of individual droplets. The data was analyzed with QuantaSoft^™^ software (Bio-Rad) based on positive and negative droplet populations. The absolute quantitation of the target DNA molecule was estimated using the Poisson statistics and presented as copies per swab in log_10_. All samples were run in duplicate.

### Study 2—diagnosis of metritis, rectal temperature, and blood sampling for immune activity analysis

To investigate the association between immune parameters and fever in metritis cows, we relied on data and samples collected for a previous study that evaluated the peripartum calcium status, energetic profile, and neutrophil function in dairy cows at low or high risk of developing uterine disease [6]. Classification of cows into MFever, MNoFever and Healthy had not been performed in the previous study. Metritis was diagnosed by examining the uterine discharge from 110 cows at 4, 7, and 12 days postpartum using a 5-point scale as previously described [6]. The farm personnel checked all cows daily from 0 to 3 DPP; therefore, any cows diagnosed with metritis and treated with antibiotics before 4 DPP were not used for this study. RT was measured daily for the first 12 DPP, and cows were classified as MFever (n = 33), MNoFever (n = 19), and Healthy (n = 58) as for the uterine microbiota analysis. Serum was prepared from whole blood collected at 0 and 3 DPP before disease onset.

#### Polymorphonuclear leukocyte (PMN) function

Function of PMN was evaluated in all 110 cows at 0 and 3 DPP to assess the potential immune response to a bacterial challenge and to avoid the effect of disease, once established, on immune response as previously described [27]. Nonetheless, it is possible that bacterial growth at 3 DPP may already affect our results. Dual-color flow cytometry (FACSort; Becton Dickinson Immunocytometry Systems, San Jose, CA) was used to gate PMN and evaluate phagocytic and oxidative burst activities after addition of a 50-μM dihydrorhodamine 123 solution and a challenge with heat-inactivated, propidium iodide-labeled *Escherichia coli*. Each sample had a negative control tube containing dihydrorhodamine 123-loaded leukocytes in blood without bacteria and a positive control tube containing phorbol 12-myristate 13-acetate. Parameters quantified from the density and fluorescence cytograms (CellQuest, version 3.3; Becton Dickinson Immunocytometry Systems, San Jose, CA) included the percentage of PMN that contained red fluorescence, indicating phagocytosis of propidium iodide-labeled *E*. *coli*, and the percentage of PMN with red and green fluorescence, indicating oxidative burst.

#### Enzyme-linked immunoassay (ELISA)

The production of TNFα and PGE_2_ was evaluated in serum samples randomly collected at 0 and 3 DPP from the Healthy (n = 14), MNoFever (n = 14), and MFever (n = 14). The production of TNFα was tested using the RayBio^®^ Bovine TNF-alpha ELISA kit (RayBiotech, Inc., Norcross, GA, USA) and the production of PGE_2_ was evaluated using the PGE Metabolite ELISA Kit (Cayman Chemical, Ann Arbor, Michigan, USA) and PGE_2_ ELISA kit—Monoclonal (Cayman Chemical) according to the manufacturer’s instructions.

### Metabolites

Serum from blood collected at 0, 2, and 4 DPP from all 202 cows (MFever = 56; MNoFever = 48; Healthy = 98 from Study 1 and 2) were used in this study. Because calcium homeostasis can affect both the body temperature and immune function [6,28,29], mean total calcium in serum collected at 0, 2 and 4 DPP was compared among the groups. Total calcium was analyzed using atomic absorption spectrometry (AAnalyst 200; Perkin-Elmer Inc., Waltham, MA) as previously reported [6]. Because blood concentrations of non-esterified fatty acids (NEFA) have been associated with the inflammatory response [18,30], serum NEFA concentration was also evaluated at 4 DPP. NEFA was measured using a commercial kit (NEFA-C Kit; Wako Diagnostics Inc., Richmond, VA) as previously reported [6].

### Data analysis

The number of reads from samples was calculated after quality filtering through the Quantitative Insights into Microbial Ecology (http://qiime.sourceforge.net/). Sequences that had no corresponding barcode and correct primer sequence were excluded. The Chao1 and Shannon indices were calculated with package fossil and vegan, respectively, under the R software (http://www.r-project.org). To visualize the dissimilarity of microbial communities, principal coordinates analysis (PCoA) was performed based on the normalized values and Manhattan distance using the MG-RAST. Dissimilarity of bacterial community was calculated based on phylum level abundance of bacteria based on Bray-Curtis distance using the vegan package of the R software version 3.3.1 (R Foundation for Statistical Computing, Vienna, Austria. URL http://www.R-project.org/) in which non-parametric multivariate analysis of variance (PERMANOVA) was used to test significant difference among groups. A heat map was generated based on the Euclidean distance of the 20 most abundant species using the R software. The relative and absolute abundance of bacteria were compared among groups by ANOVA using the GLM procedure of SAS 9.3 (SAS Institute Inc., Cary, NC, USA). The GLM models included the fixed effects of metritis group (Healthy, MNoFever or MFever), risk of metritis [high (occurrence of dystocia, twins, stillbirth or retained placenta) or low (no dystocia, twins, stillbirth or retained placenta)], parity (primiparous or multiparous), subclinical hypocalcemia [yes (Ca ≤ 8.59 mg/dL within the first 3 DPP) or no (Ca > 8.59 mg/dL within the first 3 DPP)], and the interaction between metritis group and other covariates. Serum NEFA concentrations were also analyzed by ANOVA using the GLM procedure of SAS. The model for NEFA also included the fixed effect of study (1 or 2) besides the variables listed for the other GLM models. The activity of polymorphonuclear leukocyte, production of TNFα and PGE_2_ metabolite, circulation PGE_2_, and serum concentrations of Ca were analyzed by ANOVA for repeated measures using the MIXED procedure of SAS. In addition to the variable listed for the GLM models, the MIXED models included the fixed effect of time, the interaction between metritis group and time, and cow as a random effect. Differences with *P* ≤ 0.05 were considered statistically significant; differences with 0.05 < *P* ≤ 0.10 were considered as a tendency towards statistical significance.

## Results

### Sequencing results, number of reads, Chao1 and Shannon indices

**Fig 1:**
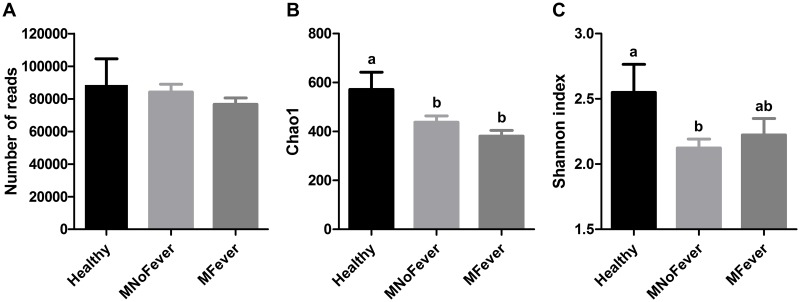
Diversity of uterine microbiota from the Healthy (n = 11), MNoFever (n = 12), and MFever (n = 11) groups. (A) Number of reads. There is no significant difference among groups. (B) Chao1 richness index. Chao1 richness estimates the total number of species in a sample based on the number of singleton and doubleton taxa. (C) Shannon diversity index. Shannon index takes into account both the species richness and evenness. Data represent the mean ± SEM. Data labeled “a” are statistically different (*P* ≤ 0.05) from data labeled “b”.

A total of 2,824,991 reads were identified in the 34 samples after quality filtering. The average number of reads was 88,405 ± 16,113, 84,147 ± 4,799, and 76,616 ± 3,957 in the Healthy, MNoFever, and MFever groups, respectively, and there was no significant difference (*P* = 0.53) among groups (Fig 1A). The Chao1 and Shannon indices were used to evaluate species richness and diversity (richness and evenness), respectively. The Chao1 index (Fig 1B) was significantly lower (*P* ≤ 0.05) in the MNoFever and MFever groups than in the Healthy group, indicating that healthy cows had a greater number of species than cows with metritis. The Shannon index (Fig 1C) was also significantly lower for the MNoFever group compared with the healthy group (2.12 ± 0.07 vs. 2.55 ± 0.21; *P* ≤ 0.05), but only numerically lower for the MFever group compared with the healthy group (2.22 ± 0.13 vs. 2.55 ± 0.21; *P* = 0.13). Taken together, the reduction in the species richness and diversity seems to be associated with metritis. However, there was no significant difference in the Chao1 (*P* = 0.37) and Shannon (*P* = 0.63) indices between the MNoFever and MFever groups, indicating that fever is not related to species richness or diversity.

### Comparisons at the whole microbiota, phylum, and genus levels

**Fig 2:**
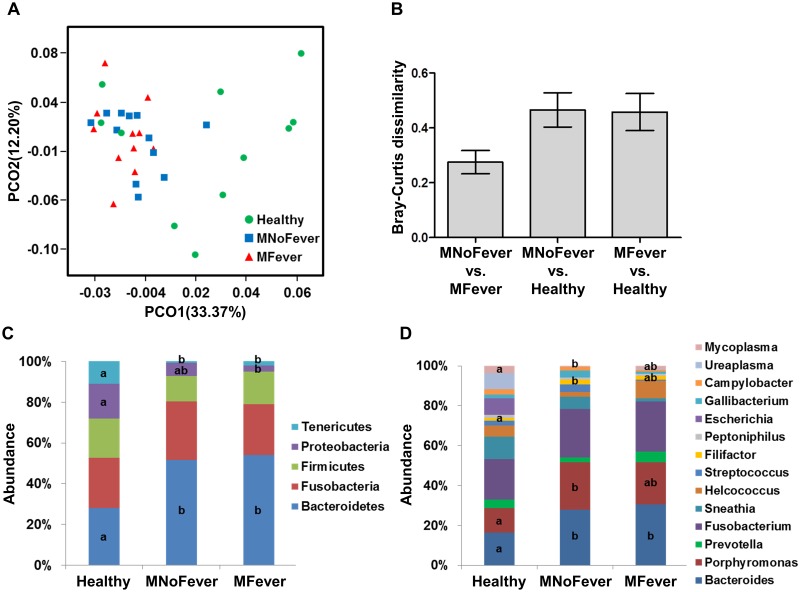
Dissimilarity of bacterial community. (A) Principal coordinates analysis of uterine microbiota based on normalized values and Manhattan distance. This shows the ordination of the Healthy (healthy cows; green circles), MNoFever (metritic cows without a fever; blue squares), and MFever (metritic cows with a fever; red triangles). Variance explained by each PCO axis is given in parentheses. (B) Bray-Curtis dissimilarity of uterine microbiota using phylum level abundance of bacteria. An index of 0 indicates the most similarity between bacterial communities, whereas an index of 1 indicates the most dissimilarity between bacterial communities. Bars represent the mean ± SEM. (C) Phylum level assignment. (D) Genus level assignment. Bacterial phyla and genera having ≥ 1% abundance were presented in a stacked bar graph. Data labeled “a” are statistically different (*P* ≤ 0.05) from data labeled “b”.

We investigated dissimilarity of uterine bacterial communities using PCoA based on normalized values and Manhattan distance (Fig 2A). The first principal component explained 33.37% of the data differences observed, which showed a separation between healthy and metritic cows. However, bacterial communities from MNoFever clustered with MFever, indicating that community separation is based on metritis but not on fever. To evaluate the degree of dissimilarity of bacterial community, we performed pairwise comparisons of samples using Bray-Curtis dissimilarity on phylum level abundance of bacteria (Fig 2B). Because pool all groups showed an average of 0.4 Bray-Curtis dissimilarity, the 0.4 index was used as benchmark to determine the difference between groups. The average of Bray-Curtis dissimilarity was 0.46 ± 0.06 between the MNoFever and Healthy groups (PERMANOVA; sequential Bonferroni significance *P* = 0.02) and 0.46 ± 0.07 between the MFever and Healthy groups (PERMANOVA; *P* = 0.01), suggesting a significant difference in bacterial communities between healthy and metritic cows. However, the MNoFever and MFever groups appeared fairly similar with an average of 0.27 ± 0.04 Bray-Curtis dissimilarity (PERMANOVA, *P* = 0.61). In agreement with the PCoA and the Bray-Curtis dissimilarity index, the uterine microbiota of MNoFever and MFever cows was strikingly similar; there was no difference (*P* > 0.25) in the major phyla (Fig 2C) or genera (Fig 2D). Metritic cows showed a greater prevalence of Bacteroidetes, and *Bacteroides* and *Porphyromonas* were the largest contributors to that difference (Fig 2C and 2D, S1 Text).

### Comparisons at the species level

**Fig 3:**
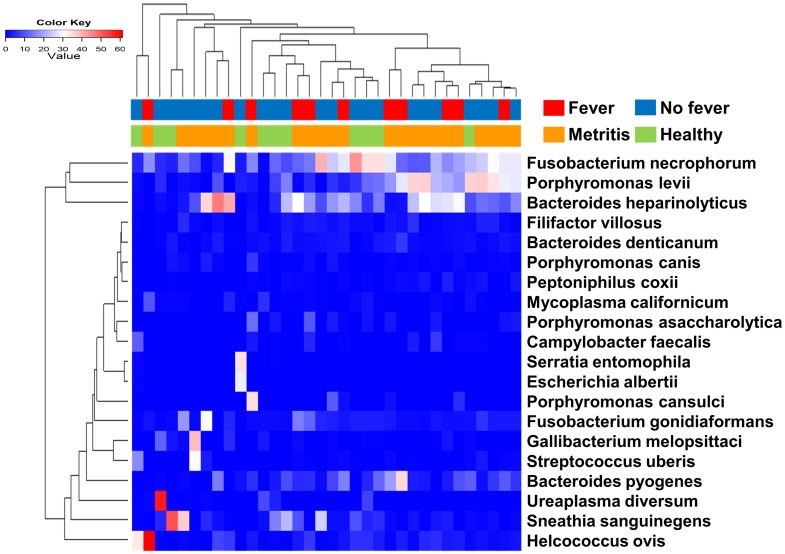
Heat map analysis with the dendrogram based on Euclidean distance. Columns represent individual cows and rows represent 20 bacterial species. The relative abundance of bacteria was presented by color in each cell. Under the dendrogram the first bar shows whether cows had fever and the second bar indicates whether cows had metritis.

**Fig 4:**
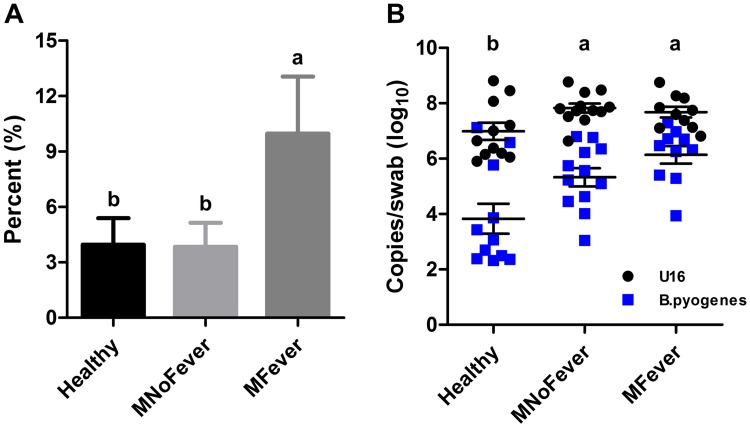
Associations of *Bacteroides pyogenes* with a fever. (A) Relative abundance of *B*. *pyogenes* based on metagenomic sequencing. Bars represent the mean ± SEM. (B) Absolute abundance of total bacteria and *B*. *pyogenes* based on droplet digital PCR. The log_10_ number of total bacteria (black circles) and *B*. *pyogenes* (blue squares) were quantified in uterine swab samples using the 16S rRNA gene and *recA* gene, respectively. Each symbol represents an individual cow and error bars indicate the means ± SEM. Data labeled “a” are statistically different (*P* ≤ 0.05) from data labeled “b”.

To identify bacterial species potentially associated with a fever, we first built a heat map based on Euclidean distance (Fig 3), but a clear cluster could not be identified for cows with metritis or with a fever. Next, we compared the relative abundance of the 35 most abundant species among groups (S4 Table). Among them, *B*. *pyogenes* was the only species that showed a significantly (*P* ≤ 0.05) higher relative abundance in the MFever group compared to the MNoFever and Healthy groups (Fig 4A).

To complement our finding, the number of total bacteria and *B*. *pyogenes* were quantified using ddPCR (Fig 4B). MNoFever (7.83 ± 0.16) and MFever groups (7.68 ± 0.19) had greater (*P* ≤ 0.05) number of total bacteria than the Healthy group (6.99 ± 0.31); however, there was no significant difference between MFever and MNoFever (*P* = 0.90), indicating that abundance of total bacteria in the uterus is related to metritis but not with fever. Likewise, the number of *B*. *pyogenes* was significantly more abundant (*P* ≤ 0.04) in the MFever (6.14 ± 0.32) and MNoFever (5.33 ± 0.33) groups than in the Healthy group (3.83 ± 0.54); however, there was no significant difference between the MFever and MNoFever groups (*P* = 0.36). It is important to notice that although no statistical difference was observed, if the sample size was increased to 33 per group and the mean differences were maintained, the comparison between MFever and MNoFever would be significant.

### Immune function

**Fig 5:**
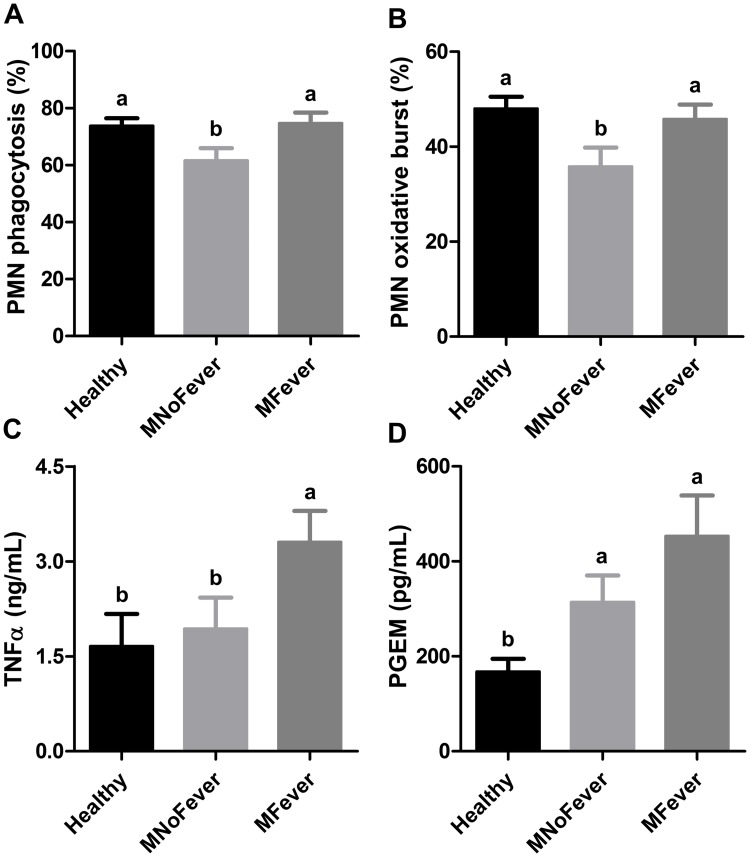
Immune function. (A) In vitro proportion of PMN with phagocytic activity (Healthy = 58, MNoFever = 19, MFever = 33). (B) In vitro proportion of PMN mediated oxidative burst (Healthy = 58, MNoFever = 19, MFever = 33). (C) In vivo serum TNFα (14 cows per group). (D) In vivo serum PGE_2_ metabolite (14 cows per group). Data represent the mean ± SEM. Data labeled “a” are statistically different (*P* ≤ 0.05) from data labeled “b”. See S1 Table for sample information.

To evaluate whether fever is associated with the immune response of cows, we measured the PMN functions in 110 cows that were classified into the Healthy, MNoFever and MFever groups using the phagocytic and oxidative burst activities. There was a main effect of group on proportion of PMN undergoing phagocytosis, with the MNoFever being lower (*P* < 0.02) than the MFever and Healthy groups (61.63 ± 4.34 vs. 74.70 ± 3.78 vs. 73.81 ± 2.66%; Fig 5A). Likewise, there was a main effect of group on proportion of PMN undergoing oxidative burst, with the MNoFever being lower (*P* ≤ 0.05) than the MFever and Healthy groups (35.81 ± 4.01 vs. 45.84 ± 3.04 vs. 48.01 ± 2.50%; Fig 5B). Although there was no interaction between group and time (*P* > 0.10), the differences between MNoFever and MFever groups seem to be more pronounced at calving (S2 Fig). The results show that PMN function is lower in the MNoFever group when compared to the MFever and Healthy groups, particularly at calving. This finding indicates inherent differences between the groups.

We evaluated the concentration of TNFα in serum, as this is one of the main pro-inflammatory cytokines involved in activation of the immune system and induction of fever. Samples (14 cows per group) were randomly chosen in 110 cows. There was a main effect of group on serum concentration of TNFα, with the MFever group being higher (*P* ≤ 0.05) than the MNoFever and Healthy groups (3.30 ± 0.49 vs. 1.94 ± 0.49 vs. 1.66 ± 0.51 ng/mL; Fig 5C). Next, on the same set of sample (14 cows per group), the concentration of PGE_2_ metabolite in serum was evaluated as a measure of PGE_2_ production indicative of endometrial response to infection [31]. There was a main effect of group on serum concentration of PGE_2_ metabolite, with the MFever and the MNoFever group being higher (*P* < 0.01) than the Healthy group (453.0 ± 85.6 vs. 314.0 ± 56.1 vs. 167.7 ± 27.1 pg/mL; Fig 5D). Despite the numerical difference, the MFever and MNoFever groups were not significantly different (*P* = 0.16). Individual data points at 0 and 3 DPP are presented in S2 Fig. Next we measured circulating PGE_2_ to evaluate the fever inducing potential. There was no difference in circulating PGE_2_ between MNoFever, MFever and Healthy groups (353.9 ± 27.6 vs. 329.6 ± 27.6 vs. 383.1 ± 28.6 pg/mL; *P* > 0.40; S3 Fig).

To find whether fever was associated with an impaired metabolic state, serum total calcium and serum NEFA concentrations were evaluated in 202 cows within 4 DPP (S4 Fig). However, both metabolites showed no difference between the MNoFever and MFever groups; both had lower calcium and higher NEFA than Healthy cows (S2 Text; S4 Fig). These data indicate that metabolic state was associated with the development of metritis, but not with the development of fever. Taken together, these data indicate that under similar metabolic conditions, metritic cows without a fever have a lower immune response than metritic cows with a fever.

## Discussion

We found that the uterine microbiota composition at the phylum and genus level, the total bacterial load, and the absolute numbers of *B*. *pyogenes* were very similar between metritic cows with and without a fever. The uterine bacterial load and community were associated with metritis but not with a fever. Similar to our recent observations in metritic cows with a fever [15], the uterine microbiota of metritic cows without a fever was dominated by the phyla Bacteroidetes and Fusobacteria. Bacterial communities from metritic cows with a fever were not distinguished from those from metritic cows without a fever on PCoA and heat map analysis. Because of our current understanding of LPS [32] which activate pyrogenic cytokines and induce fever, it was assumed that Gram-negative bacteria would be more prevalent in metritic cows with a fever than in metritic cows without a fever. However, in this study the abundance of Gram-negative bacteria was not clearly relevant to development of fever. To explore infectious agents for the development of fever, we analyzed the bacterial contribution to fever development in metritic cows using the species abundance of bacteria. The metagenomic sequencing data pointed to *B*. *pyogenes* as a potential fever inducing pathogen in metritic cows. Nonetheless, although *B*. *pyogenes* was found to be very abundant in cows with metritis, the absolute number of *B*. *pyogenes* and all bacteria were similar between the MNoFever and MFever groups. Even so, as mentioned in the results, it is important to conduct further studies with a larger sample size to definitely exclude the possibility of involvement of *B*. *pyogenes* in the development of fever in metritic cows. *B*. *pyogenes* was first identified from abscesses and feces of pigs [33], and then it has been identified from infected wounds after cat and dog bites in humans [34–36]. Only recently, *B*. *pyogenes* was identified in the uterus of dairy cows [37], and this is the first report of higher abundance in metritic cows; therefore, the pathogenicity of *B*. *pyogenes* should be further investigated. Overall pathogenicity of *Bacteroides* spp. includes the presence of a polysaccharide capsule, release of LPS, evasion of host immune response, production of proteolytic enzymes (e.g. hyaluronidase and chondroitin sulfatase), production of hemolysin, release of enterotoxin, and aerotolerance [38]. Our findings of similar bacterial community in metritic cows with or without a fever was surprising to us because fever has usually been used as an indicator of the severity of the infection [4]. This finding may have important consequences for treatment decisions. In the past, only metritic cows with a fever have been included in studies for approval of new antibiotics, which may lead producers to believe that metritis without a fever does not require antibiotic treatment [7,8]. Because the bacterial community is virtually identical between metritic cows with and without a fever, it would be appropriate to use similar antimicrobial therapy for either case.

In addition to the pathogenic etiology of fever, it was thought that the cow’s response to uterine bacteria may also be relevant to the induction of fever. We also evaluated the immune function in a large number of cows (n = 110) at 0 and 3 DPP before the cows developed metritis. Interestingly, the PMN function was significantly lower in metritic cows without a fever than in metritic cows with a fever, particularly at calving, which likely affects bacterial clearance and disease development. Therefore, impaired immune function at the day of calving possibly affects development of metritis and may indicate the ability to induce a fever. However, a more suppressed immune function in metritic cows without a fever lay in contrast with the reported higher clinical cure rate after treatment in metritic cows without a fever than in metritic cows with a fever [10]. Nonetheless, a more robust inflammatory response may lead to more tissue damage because of generation and release of reactive oxygen species and the release of proteolytic enzymes [39], which may delay healing and elimination of clinical signs. A recent study reported a greater suppression of fertility in metritic cows with a fever than in metritic cows without a fever, which may be another indication of greater tissue damage in metritic cows with a fever [9]. Lesser tissue damage during the metritis event may allow metric cows without a fever to heal and recover fertility quicker after antibiotic treatment than metritic cows with a fever [9,10]. Therefore, metritis with a fever may indicate a worse case of disease but not because of differences in the microbiome but because of differences in the immune response to the bacteria in the uterus, and the associated damage to the uterus.

We measured TNFα, to try to understand the differences in immune function and get insights into the fever inducing potential in metritic cows. TNFα is a major pro-inflammatory cytokine involved in activation of professional phagocytes and induction of a febrile response [12]. After contact with bacteria, tissue macrophages are stimulated to produce and release pro-inflammatory cytokines and chemokines, including TNFα, IL-1β, IL-6, and IL-8, which stimulate neutrophil and monocyte diapedesis and chemoattraction, promote increased phagocytosis and bacterial killing, and induce a febrile response [12,40]. Pro-inflammatory cytokines lead to a febrile response by inducing the production of PGE_2_ by brain vessels or release of PGE_2_ by Kupffer cells in the liver, which stimulate vagal afferent nerves that transmit the pyrogenic message to the preoptic anterior hypothalamic area that control body temperature [12,41]. After infection, the endometrial cells of the uterus produce and release PGE_2_ [31]; therefore, we measured PGE_2_ and PGE_2_ metabolite to evaluate if production of PGE_2_ in the uterus could contribute to the febrile response. Rapid metabolism of PGE_2_ in the lungs probably eliminated differences in circulating PGE_2_ among the groups. Similar concentrations of PGE_2_ metabolite between metritic cows with and without a fever indicates similar capacity to produce PGE_2_. Nonetheless, it is possible that cytokine-induced local production of PGE_2_ in the brain or in the liver may be more important for induction of a fever in metritic cows than uterine production of PGE_2_. The source of TNFα is probably tissue macrophages and activated monocytes and PMN because endometrial cells do not secrete appreciable amounts of TNFα [42]. Therefore, our data indicate that metritic cows without a fever have an inherent lower inflammatory response with lower PMN function and lower production of TNFα despite a similar bacterial challenge to metritic cows with a fever. It is not clear why metritic cows without a fever have a lower inflammatory response; therefore, factors involved in the initiation of the inflammatory cascade such as toll-like receptors, nuclear factor-κB and others should be further investigated.

We also explored the contribution of calcium and NEFA blood concentrations, as these metabolites had been found to be associated with PMN function in previous studies [6,18]. Nonetheless, both metabolites were remarkably similar between metritic cows with and without a fever, which did not help explain the lower PMN activity in metric cows without a fever. A downside of our study is that the microbiome and immune parameters were evaluated in two different samples from the same population of cows. Whereas there may be little difference in the microbial composition between MFever and MNoFever groups, there could still be a correlation between specific immune parameters and specific phyla, genera, or species of bacteria in the individual animals. In the future, both the microbial component and the immune component should be evaluated in the same animals.

In summary, we explored both infectious agents and host factors that may trigger a fever during uterine infections. Our study is the first to show a similar microbiome between metritic cows with and without a fever, which indicates that the host response may be more important for fever development than the microbiome. *Bacteroides pyogenes* was identified as an important pathogen for the development of metritis but not fever. The decreased inflammatory response characterized by decreased PMN activity and cytokine production in the MNoFever group may explain the lack of a febrile response.

## Supporting Information

S1 Text Comparisons at the phylum and genus levels. (PDF)

S2 Text Metabolic state. (PDF)

S1 Fig Rectal temperature of the Healthy, MNoFever, and MFever groups from Study 1. On the day of metritis diagnosis (0 days), the average RT was 39.0°C ± 0.1, 39.1°C ± 0.1, and 39.9°C ± 0.1 in the Healthy, MNoFever, and MFever groups, respectively. Data represent mean ± SEM and an asterisk indicates statistical significance at *P < 0.05 and **P < 0.01.
(TIF)

S2 Fig Immune function in the Healthy, MNoFever, and MFever groups at 0 and 3 days postpartum. (A) In vitro proportion of PMN with phagocytic activity (Healthy = 58, MNoFever = 19, MFever = 33). There was a main effect of group on proportion of PMN undergoing phagocytosis, with the MNoFever being lower (*P* < 0.02) than the MFever and Healthy groups. (B) In vitro proportion of PMN mediated oxidative burst (Healthy = 58, MNoFever = 19, MFever = 33). There was a main effect of group on proportion of PMN undergoing oxidative burst, with the MNoFever being lower (*P* < 0.05) than the MFever and Healthy groups. (C) In vivo serum TNFα (14 cows per group). There was a main effect of group on serum concentration of TNFα. There was a main effect of group on serum concentration of TNFα, with the MFever group being higher (*P* ≤ 0.05) than the MNoFever and Healthy groups. (D) In vivo serum PGE_2_ metabolite (14 cows per group). There was a main effect of group on serum concentration of PGE2 metabolite, with the MFever and the MNoFever group being higher (*P* < 0.01) than the Healthy group. Data were analyzed by ANOVA for repeated measures using the MIXED procedure of SAS that includes the fixed effect of time, the interaction between metritis group and time, and cow as a random effect. The interaction between metritis group and time was not significant (*P* > 0.10) in all analyses.
(TIF)

S3 Fig The production of circulating PGE_2_. The production of PGE_2_ was evaluated in serum using the PGE_2_ ELISA kit—Monoclonal (Cayman Chemical) according to the manufacturer’s instructions. Data represent mean ± SEM. There was no significant difference among groups.
(TIF)

S4 Fig Metabolic parameters. (A) serum calcium (Ca) concentration at 0, 2, and 4 days postpartum and (B) serum concentration of non-esterified fatty acids (NEFA) at 4 days postpartum were measured in the Healthy, MNoFever, and MFever groups. Data represent mean ± SEM. Small letters indicate statistical significance at *P* ≤ 0.05 and capital letters indicate tendency to significance at 0.05 < *P* < 0.10.
(TIF)

S1 Table Overview of cows enrolled and used in the study. (PDF)

S2 Table Metadata for study 1 and study 2. (PDF)

S3 Table MG-RAST IDs. (PDF)

S4 Table Relative abundance of the 35 most abundant species. (PDF)
