# Supplementary material for: Uterine Microbiota and Immune Parameters Associated with Fever in Dairy Cows with Metritis
Source: PLoS One. 2016 Nov 1;11(11):e0165740. doi: 10.1371/journal.pone.0165740 (PMC5089738; doi:10.1371/journal.pone.0165740)
Supplement: S1 Text — (PDF) [file pone.0165740.s001.pdf]

S1 Text Comparisons at the phylum and genus levels

To identify differences in uterine microbiota by fever, we examined the relative abundance of bacterial phyla and genera having ≥ 1% of abundance among groups (S2 Fig). Twenty-five phyla were detected in the uterus of all dairy cows. Bacteroidetes (44.1%), Fusobacteria (25.8%), Firmicutes (15.5%), Proteobacteria (8.6%), and Tenericutes (4.3%) were the five most abundant bacterial phyla, which accounted for 98.3% of the bacterial community. Bacteroidetes was significantly higher ($P < 0.01$) for MNoFever and MFever groups than the Healthy group (51.1 vs. 53.3 vs. 27.3%, respectively). Tenericutes was significantly lower ($P < 0.04$) for MNoFever and MFever groups than the Healthy group (0.7 vs. 2.0 vs. 10.6%, respectively). Proteobacteria was lower for the MFever compared with the Healthy group (2.9 vs. 16.6%; $P \leq 0.05$), but it was statistically similar for the MNoFever compared with the Healthy group (6.5 vs. 16.6%; $P = 0.13$). Meanwhile, there was no difference ($P > 0.5$) in the relative abundance of the major phyla between MNoFever and MFever groups.

At the genus level, of 665 genera, *Bacteroides* (22.4%) was the most abundant genus in all samples, followed by *Fusobacterium* (20.8%), *Porphyromonas* (17.4%), *Sneathia* (5.3%), *Helcococcus* (4.7%), *Prevotella* (3.4%), *Streptococcus* (2.1%), *Escherichia* (2.1%), *Ureaplasma* (2.0%), *Gallibacterium* (1.9%), *Mycoplasma* (1.6%), *Filifactor* (1.6%), *Campylobacter* (1.6%), and *Peptoniphilus* (1.1%). Genus-level assignments showed a higher ($P \leq 0.05$) proportion of *Bacteroides* and *Porphyromonas* in the MNoFever (26.1% and 22.5%, respectively) and MFever (28.3% and 19.7%, respectively) than in the Healthy group (12.5% and 9.6%, respectively), indicating that the genus *Bacteroides* and *Porphyromonas* are attributed to high abundance of Bacteroidetes in metritic cows. In addition, the MNoFever group had lower abundance of *Mycoplasma* ($P = 0.03$) and higher abundance of *Filifactor* ($P = 0.03$) compared with the Healthy group. Similar to PCoA and comparisons at the phylum level, however, there was no difference ($P > 0.25$) in the relative abundance of the major genera between MNoFever and MFever groups. All data indicated a high degree of similarity between the uterine microbiota of metritic cows with and without a fever.
