# Supplementary material for: Uterine Microbiota and Immune Parameters Associated with Fever in Dairy Cows with Metritis
Source: PLoS One. 2016 Nov 1;11(11):e0165740. doi: 10.1371/journal.pone.0165740 (PMC5089738; doi:10.1371/journal.pone.0165740)
Supplement: S2 Text — (PDF) [file pone.0165740.s002.pdf]

## S2 Text Metabolic state

To find whether fever was associated with an impaired metabolic state, serum total calcium and serum NEFA concentrations were evaluated in 202 cows within 4 DPP (S3 Fig). Serum total calcium concentration was similar among groups at 0 DPP, but cows with metritis dropped significantly at 2 DPP. The MNoFever and MFever had lower ($P < 0.01$) serum total calcium concentration than the Healthy group at 2 DPP (7.9 ± 0.2 vs. 8.1 ± 0.1 vs. 8.5 ± 0.1 mg/dL) and 4 DPP (8.3 ± 0.2 vs. 8.2 ± 0.1 vs. 8.9 ± 0.1 mg/dL). On the other hand, the concentration of NEFA tended to be higher ($P < 0.1$) in the MNoFever and MFever groups than in the Healthy group (803.3 ± 73.9 vs. 801.0 ± 73.2 vs. 657.7 ± 45.5 µEq/L). Both metabolites showed no difference between the MNoFever and MFever groups. These data indicate that metabolic state was associated with the development of metritis, but it was not related to a fever.
