# Supplementary figures and images for: Uterine Microbiota and Immune Parameters Associated with Fever in Dairy Cows with Metritis
Source: PLoS One. 2016 Nov 1;11(11):e0165740. doi: 10.1371/journal.pone.0165740 (PMC5089738; doi:10.1371/journal.pone.0165740)

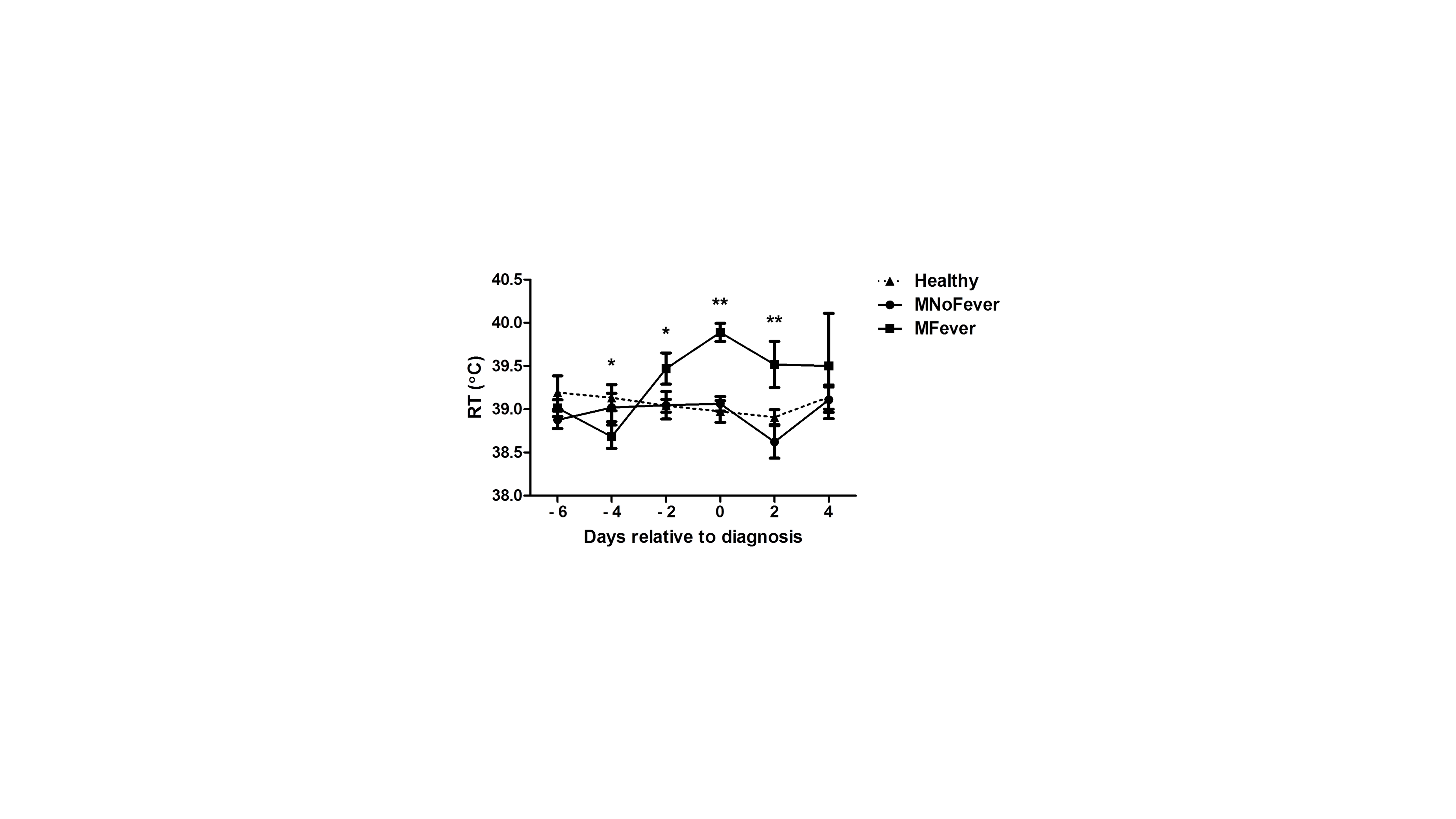

Supplement: S1 Fig — On the day of metritis diagnosis (0 days), the average RT was 39.0°C ± 0.1, 39.1°C ± 0.1, and 39.9°C ± 0.1 in the Healthy, MNoFever, and MFever groups, respectively. Data represent mean ± SEM and an asterisk indicates statistical significance at *P < 0.05 and **P < 0.01. (TIF) [file pone.0165740.s003.tif]

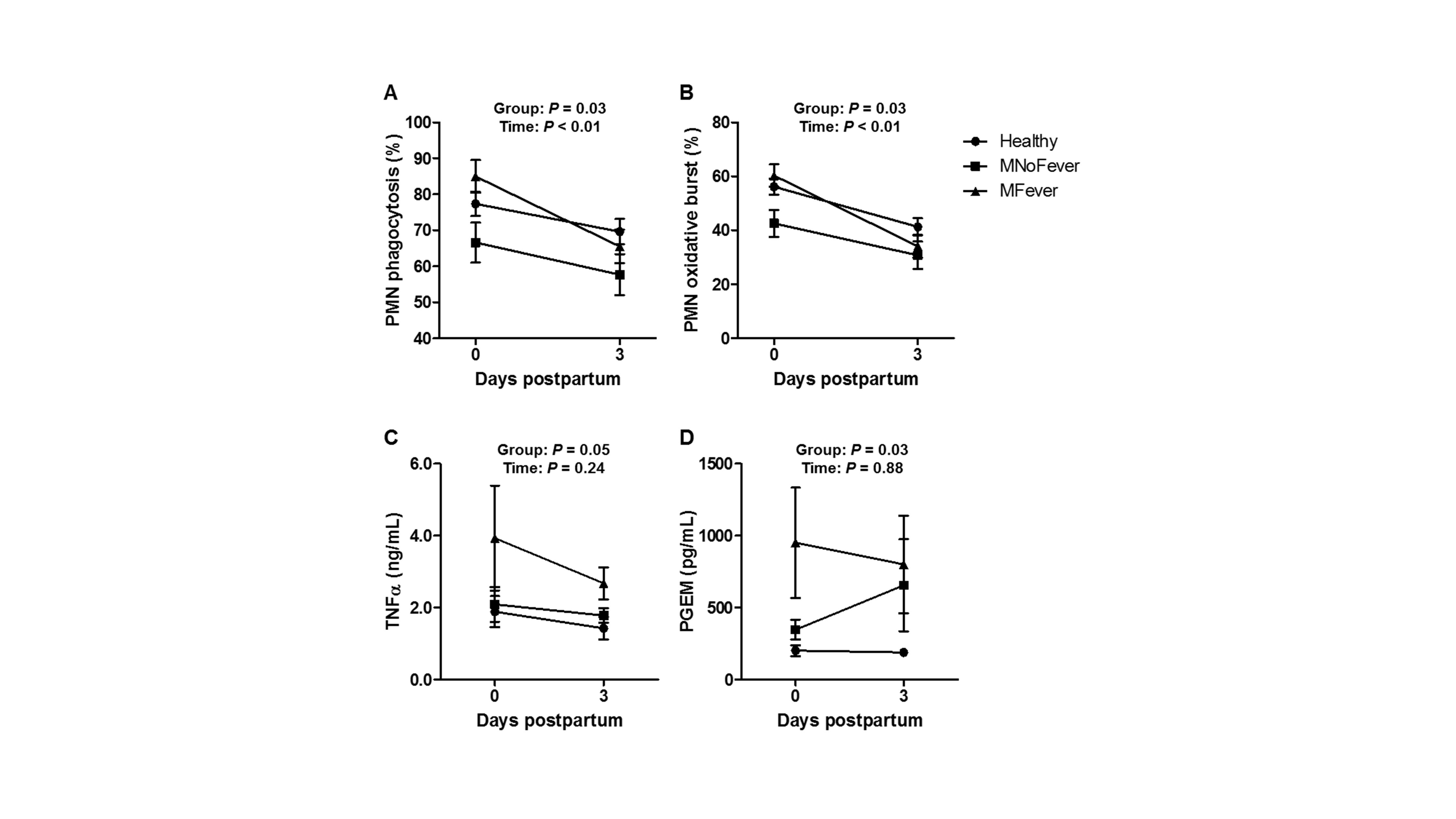

Supplement: S2 Fig — (A) In vitro proportion of PMN with phagocytic activity (Healthy = 58, MNoFever = 19, MFever = 33). There was a main effect of group on proportion of PMN undergoing phagocytosis, with the MNoFever being lower (P < 0.02) than the MFever and Healthy groups. (B) In vitro proportion of PMN mediated oxidative burst (Healthy = 58, MNoFever = 19, MFever = 33). There was a main effect of group on proportion of PMN undergoing oxidative burst, with the MNoFever being lower (P < 0.05) than the MFever and Healthy groups. (C) In vivo serum TNFα (14 cows per group). There was a main effect of group on serum concentration of TNFα. There was a main effect of group on serum concentration of TNFα, with the MFever group being higher (P ≤ 0.05) than the MNoFever and Healthy groups. (D) In vivo serum PGE2 metabolite (14 cows per group). There was a main effect of group on serum concentration of PGE2 metabolite, with the MFever and the MNoFever group being higher (P < 0.01) than the Healthy group. Data were analyzed by ANOVA for repeated measures using the MIXED procedure of SAS that includes the fixed effect of time, the interaction between metritis group and time, and cow as a random effect. The interaction between metritis group and time was not significant (P > 0.10) in all analyses. (TIF) [file pone.0165740.s004.tif]

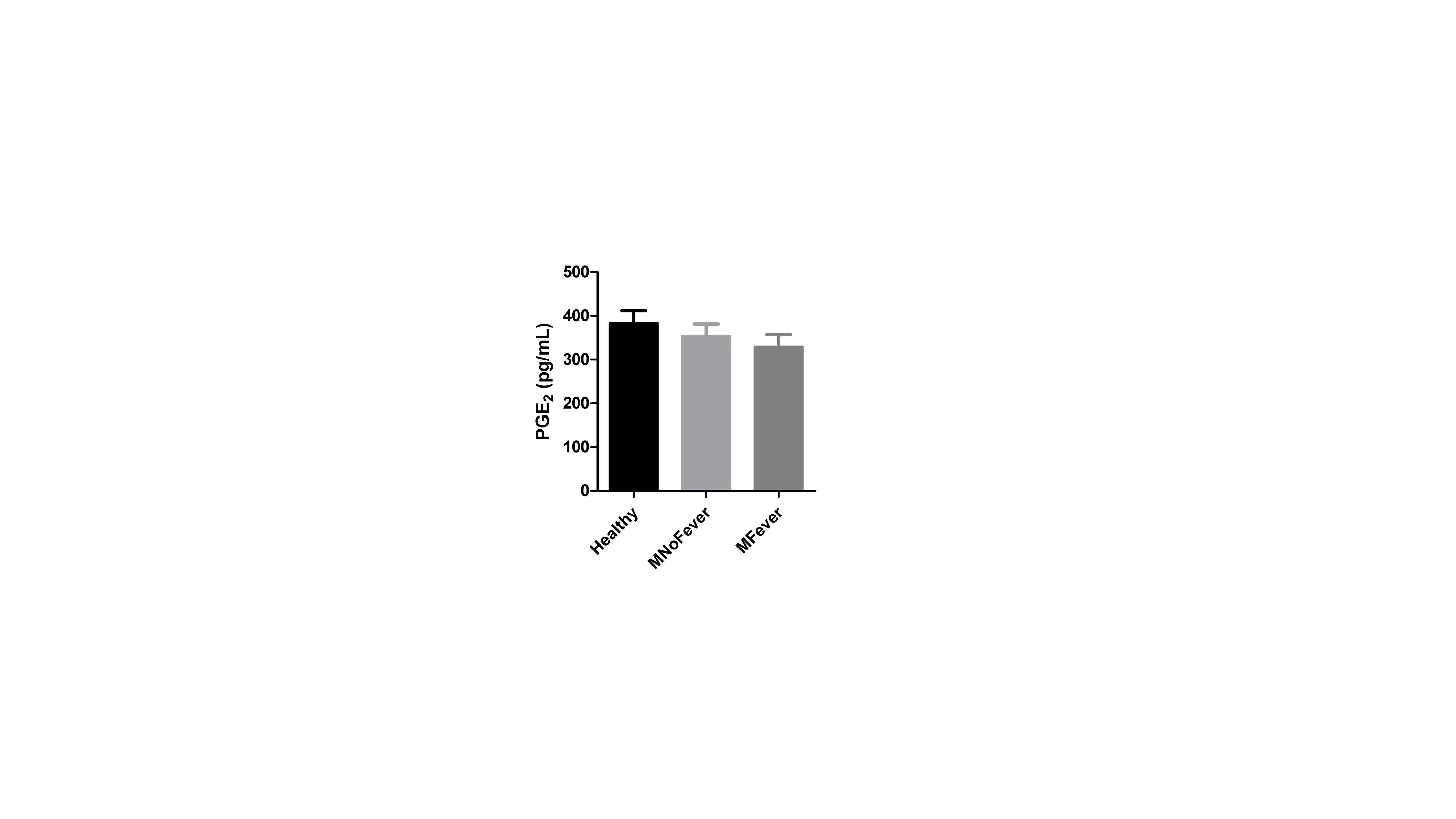

Supplement: S3 Fig — The production of PGE2 was evaluated in serum using the PGE2 ELISA kit—Monoclonal (Cayman Chemical) according to the manufacturer’s instructions. Data represent mean ± SEM. There was no significant difference among groups. (TIF) [file pone.0165740.s005.tif]

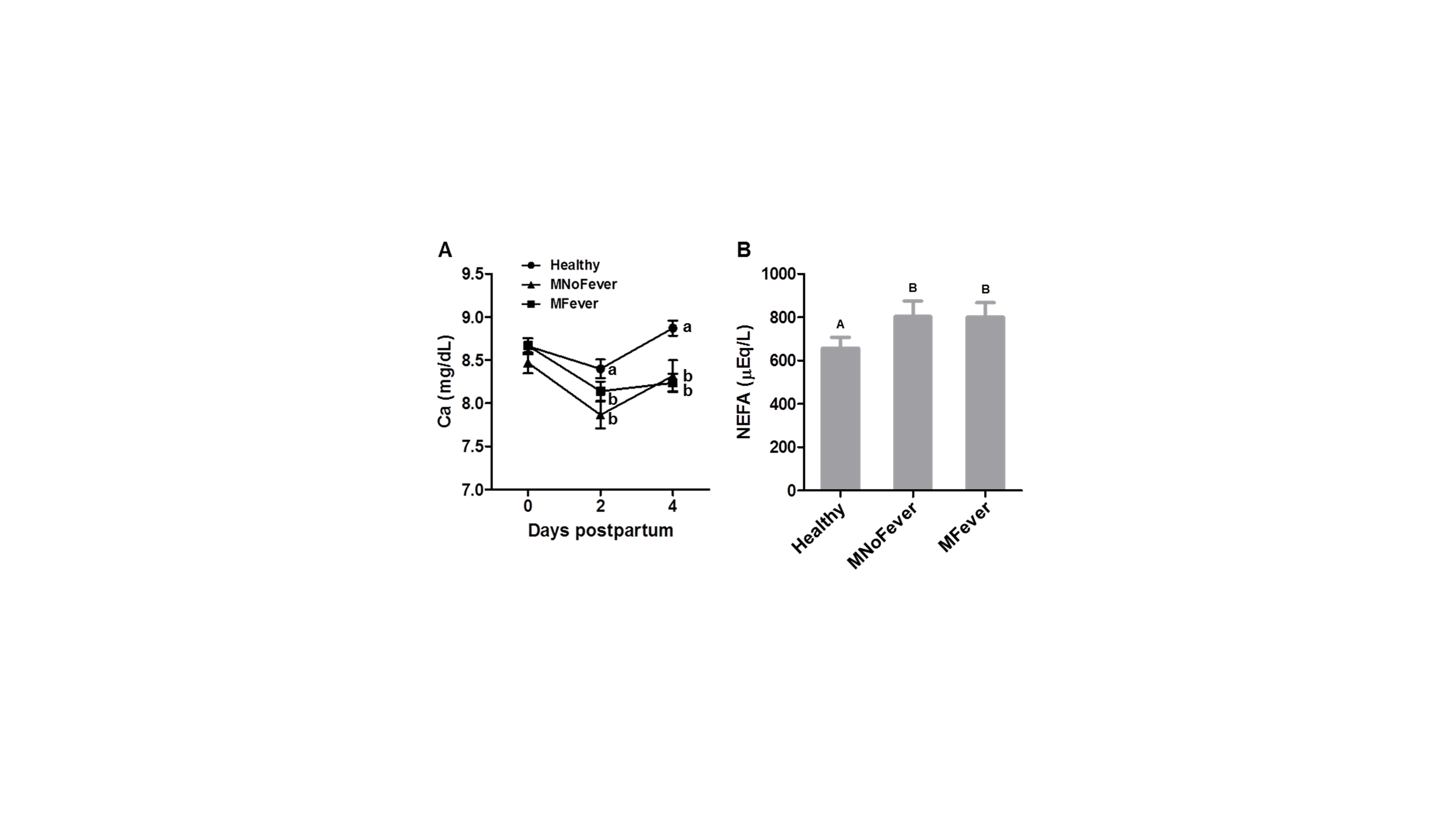

Supplement: S4 Fig — (A) serum calcium (Ca) concentration at 0, 2, and 4 days postpartum and (B) serum concentration of non-esterified fatty acids (NEFA) at 4 days postpartum were measured in the Healthy, MNoFever, and MFever groups. Data represent mean ± SEM. Small letters indicate statistical significance at P ≤ 0.05 and capital letters indicate tendency to significance at 0.05 < P < 0.10. (TIF) [file pone.0165740.s006.tif]
