# Supplementary material for: Uterine Microbiota and Immune Parameters Associated with Fever in Dairy Cows with Metritis
Source: PLoS One. 2016 Nov 1;11(11):e0165740. doi: 10.1371/journal.pone.0165740 (PMC5089738; doi:10.1371/journal.pone.0165740)
Supplement: S1 Table — (PDF) [file pone.0165740.s007.pdf]

S1 Table. Overview of cows and samples used in the study.

	Study 1	Study 2		Study 1 and Study 2
Purpose	Uterine microbiota	Immune function		Metabolic state
No. of cows enrolled	92	110		202
Day of sampling*	6 ± 2 DPP	0 and 3 DPP		0, 2, and 4 DPP
No. of samples for analysis	Metagenomics, ddPCR	PMN activity	TNFα, PGE_2	Ca, NEFA
Healthy	11	58	14	98
MNoFever	12	19	14	48
MFever	11	33	14	56

***** Samples for Study 1 were collected at the day of disease diagnosis and samples for Study 2 were collected before disease onset. DPP = days postpartum.
