# Supplementary material for: Uterine Microbiota and Immune Parameters Associated with Fever in Dairy Cows with Metritis
Source: PLoS One. 2016 Nov 1;11(11):e0165740. doi: 10.1371/journal.pone.0165740 (PMC5089738; doi:10.1371/journal.pone.0165740)
Supplement: S2 Table — (PDF) [file pone.0165740.s008.pdf]

S2 Table. Metadata for study 1 and study 2. These risk factors were included as the variable in the GLM and MIXED models.

	Healthy		MNoFever		MFever	
n (%)	Study 1	Study 2	Study 1	Study 2	Study 1	Study 2
Parity						
Primiparous	4 (36.4)	14 (24.1)	2 (16.7)	8 (42.1)	7 (63.6)	14 (42.4)
Multiparous	7 (63.6)	44 (75.9)	10 (83.3)	11 (57.9)	4 (36.4)	19 (57.6)
Dystocia	1 (9.1)	12 (20.7)	6 (50.0)	8 (42.1)	3 (27.3)	14 (42.4)
Twins	0 (0.0)	3 (5.2)	0 (0.0)	2 (10.5)	0 (0.0)	8 (24.2)
Stillbirth	0 (0.0)	3 (5.2)	0 (0.0)	1 (5.3)	2 (18.2)	3 (9.1)
Retained placenta	0 (0.0)	3 (5.2)	1 (8.3)	3 (15.8)	2 (18.2)	10 (30.3)
Subclinical hypocalcemia	4 (36.0)	26 (44.8)	9 (75.0)	14 (73.7)	6 (54.5)	32 (97.0)
