# Supplementary material for: Uterine Microbiota and Immune Parameters Associated with Fever in Dairy Cows with Metritis
Source: PLoS One. 2016 Nov 1;11(11):e0165740. doi: 10.1371/journal.pone.0165740 (PMC5089738; doi:10.1371/journal.pone.0165740)
Supplement: S3 Table — (PDF) [file pone.0165740.s009.pdf]

S3 Table. MG-RAST IDs.

Sample No.	Animal No.	Study group	Days postpartum	MG-RAST ID
SJ18	12437	MFever	8	4584525
SJ21	12517	MFever	4	4584526
SJ24	12375	MFever	6	4584527
SJ27	10955	MFever	8	4584528
SJ30	12258	MFever	6	4584529
SJ69	12484	MFever	4	4584542
SJ75	12279	MFever	6	4584544
SJ78	13302	MFever	8	4584545
SJ81	28679	MFever	8	4584546
SJ84	202	MFever	8	4584547
SJ87	9685	MFever	6	4584548
SJ33	14918	MNoFever	6	4584530
SJ36	4512	MNoFever	4	4584531
SJ39	9089	MNoFever	6	4584532
SJ42	13776	MNoFever	6	4584534
SJ45	8277	MNoFever	6	4584535
SJ88	9333	MNoFever	4	4584549
SJ89	10212	MNoFever	4	4584550
SJ90	2688	MNoFever	6	4584551
SJ91	438	MNoFever	8	4584552
SJ92	12414	MNoFever	6	4584553
SJ93	19328	MNoFever	6	4584554
SJ94	4853	MNoFever	8	4584555
SJ3	9854	Healthy	8	4584533
SJ6	8085	Healthy	4	4584543
SJ9	15527	Healthy	6	4584556
SJ12	265	Healthy	8	4584523
SJ15	16018	Healthy	6	4584524
SJ48	10514	Healthy	4	4584536
SJ54	6870	Healthy	6	4584537
SJ57	246	Healthy	8	4584538
SJ60	9284	Healthy	8	4584539
SJ63	12265	Healthy	6	4584540
SJ66	10991	Healthy	8	4584541
