# Supplementary material for: Uterine Microbiota and Immune Parameters Associated with Fever in Dairy Cows with Metritis
Source: PLoS One. 2016 Nov 1;11(11):e0165740. doi: 10.1371/journal.pone.0165740 (PMC5089738; doi:10.1371/journal.pone.0165740)
Supplement: S4 Table — (PDF) [file pone.0165740.s010.pdf]

S4 Table. Relative abundance of the 35 most abundant species.

	Relative abundance (%)			
**Species**	**All**	**Healthy**	**MNoFever**	**MFever**
Fusobacterium necrophorum	18.1	15.8	18.2	20.3
Bacteroides heparinolyticus	14.9	**7.0[a,A]**	**20.3[b]**	16.8[B]
Porphyromonas levii	13.0	9.4	17.7	11.4
Sneathia sanguinegens	6.2	10.6[A]	6.6[A,B]	1.5[B]
Bacteroides pyogenes	5.9	**4.0[a]**	**3.9[a]**	**10.0[b]**
Helcococcus ovis	5.0	4.6	2.2	8.6
Fusobacterium gonidiaformans	4.2	1.7	6.3	4.4
Ureaplasma diversum	2.4	7.2	0.1	0.1
Gallibacterium melopsittaci	2.0	1.7	3.4	0.9
Streptococcus uberis	1.8	1.7	3.2	0.4
Filifactor villosus	1.6	**0.9[a]**	**2.3[b]**	1.7[a,b]
Porphyromonas cansulci	1.6	0.02	0.9	4.0
Bacteroides denticanum	1.6	1.4	1.3	2.1
Porphyromonas asaccharolytica	1.3	0.5	0.8	2.6
Serratia entomophila	1.1	3.3	0.03	0.02
Mycoplasma californicum	1.0	1.3	0.2	1.5
Campylobacter faecalis	1.0	1.1	0.9	0.8
Escherichia albertii	0.9	2.7	0.02	0.01
Porphyromonas canis	0.9	0.5	1.0	1.1
Peptoniphilus coxii	0.7	0.4	0.8	0.9
Escherichia coli	0.7	2.0	0.01	0.01
Candidatus Blochmannia rufipes	0.7	0.8	0.7	0.4
Bacteroides zoogleoformans	0.7	**0.3[a,A]**	**1.0[b]**	0.7[B]
Mycoplasma canadense	0.6	1.7	0.05	0.2
Prevotella buccalis	0.5	0.3	0.8	0.4
Porphyromonas somerae	0.5	0.7	0.6	0.2
Peptoniphilus indolicus	0.5	1.0	0.2	0.3
Prevotella amnii	0.4	0.2[a,b]	**0.1[a]**	**1.0[b]**
Veillonella magna	0.4	0.4	0.4	0.4
Prevotella albensis	0.4	**0.6[a]**	**0.04[b,B]**	0.5[A]
Peptostreptococcus anaerobius	0.4	0.3	0.3	0.5
Porphyromonas endodontalis	0.4	0.04	0.01	1.0
Trueperella pyogenes	0.3	0.1	0.3	0.6
Campylobacter mucosalis	0.3	0.8	0.2	0.1
Prevotella paludivivens	0.3	0.4[a,b]	**0.05[a]**	**0.6[b]**

[a,b]Superscripts within a row are significantly different; $P \leq 0.05$. [A,B]Superscripts within a row tend to differ; $0.05 < P < 0.10$.
